# Employment Condition, Economic Deprivation and Self-Evaluated Health in Europe: Evidence from EU-SILC 2009–2012

**DOI:** 10.3390/ijerph14020143

**Published:** 2017-02-03

**Authors:** Silvia Bacci, Claudia Pigini, Marco Seracini, Liliana Minelli

**Affiliations:** 1 Department of Economics, University of Perugia (IT), 06123 Perugia, Italy; marco.seracini@unipg.it; 2 Department of Economics and Social Sciences, Università Politecnica delle Marche (IT), 60121 Ancona, Italy; c.pigini@univpm.it; 3 Department of Experimental Medicine, University of Perugia (IT), 06123 Perugia, Italy; liliana.minelli@unipg.it

**Keywords:** correlated random-effects model, economic deprivation, longitudinal data, ordered logit model, part-time, self-evaluated health status, temporary employment, unemployment

## Abstract

*Background*: The mixed empirical evidence about employment conditions (i.e., permanent vs. temporary job, full-time vs. part-time job) as well as unemployment has motivated the development of conceptual models with the aim of assessing the pathways leading to effects of employment status on health. Alongside physically and psychologically riskier working conditions, one channel stems in the possibly severe economic deprivation faced by temporary workers. We investigate whether economic deprivation is able to partly capture the effect of employment status on Self-evaluated Health Status (SHS). *Methods*: Our analysis is based on the European Union Statistics on Income and Living Conditions (EU-SILC) survey, for a balanced sample from 26 countries from 2009 to 2012. We estimate a correlated random-effects logit model for the SHS that accounts for the ordered nature of the dependent variable and the longitudinal structure of the data. *Results and Discussion*: Material deprivation and economic strain are able to partly account for the negative effects on SHS from precarious and part-time employment as well as from unemployment that, however, exhibits a significant independent negative association with SHS. *Conclusions*: Some of the indicators used to proxy economic deprivation are significant predictors of SHS and their correlation with the employment condition is such that it should not be neglected in empirical analysis, when available and further to the monetary income.

## 1. Background

A prominent amount of theoretical and empirical literature has focused on the relationship between workers’ employment condition and their well-being. Special attention has been devoted to the adverse health effects of unemployment [1] and, more recently, to understanding precarious employment and underemployment as an emerging social determinant of health [2,3]. These issues are particularly relevant in the context of the latest economic downturn, leading to an EU-28 unemployment rate of about 20% in 2015 and to an incidence of temporary employment of 40% for workers aged between 14 and 25 in 2014. Furthermore, in the same category, over 19% works less than 20 h per week [4].

Unemployment has long been found to be associated with detrimental effects on several health outcomes, especially through the engagement in riskier health-related life-styles, following the experience of a stressful life event, and/or through prolonged spells of economic deprivation. Consequences range from poorer Self-evaluated Health Status (SHS henceforth) [5,6], mental illness [7,8], higher incidence of cardio-vascular diseases [9] and all-cause mortality [10]. In addition, long-term unemployment exacerbates the negative health effects of unemployment [11] and is associated with a higher incidence of suicide [12].

More recently, several studies have also recognized temporary employment to exert a negative effect on health. The channels describing this negative relationship have been identified with the structural insecurity related to contract and income instability and with worse (psychological) working conditions, in terms of a lower degree of employment protection, compared to workers with open-ended contracts. In particular, in Spain temporary workers experience disempowerment in bargaining over wages and working hours and a low degree entitlement to workplace rights [13]. Potential economic deprivation resulting from intermittent spells of employment, insecurity about contract duration, and the harsher psychological working environment faced by temporary workers have been found to have detrimental effects on SHS in several countries, such as South Korea [14], Denmark [15], and Japan [16], and to be associated with a lower psychological well-being (see, for instance, [17] for a study on Sweden) and poor mental health [18].

Another form of underemployment, which has also been found to exert detrimental effects on the workers’ SHS and psychological well-being, is represented by the working time effort [3,19] that entails differences in SHS between part-time and full-time employees, especially in terms of unmet financial needs. In particular, working less hours compared to having a full-time contract has been found to be associated with poor mental health, depression and with the engagement in unhealthy behaviors [20,21].

In this framework, it is relevant to identify the extent to which the negative effects of temporary and part-time employment, as well as unemployment, are channeled by a state of economic strain, whose consequences may be alleviated by an adequate welfare supporting system, independently from contract-specific effects related to the structural and perceived hardship of the work environment.

In the present work, we investigate whether economic deprivation, intended mainly in terms of material deprivation and economic strain, is able to capture the negative effect of precarious and part-time employment, other than that of unemployment, on SHS. Our analysis is based on the longitudinal European Union Statistics on Income and Living Conditions (EU-SILC) survey conducted by Eurostat, of which we consider the sample of citizens from 26 European countries entered in the study in 2009 and interviewed for four consecutive waves until 2012. The EU-SILC survey contains a rich section on economic deprivation, which provides us with information on the households’ material deprivation, in terms of their ability to face ordinary/unexpected expenses and bear loan repayments, afford desirable and necessary items for everyday living, and to maintain adequate housing conditions, and economic strain. These measures are more representative of the perceived economic condition than monetary household income, often employed as a proxy for deprivation in analyzing the relationship between employment condition and SHS. We estimate a correlated random-effects logit model for the SHS that accounts for the ordered nature of the dependent variable and the longitudinal structure of the data. Although its subjective nature, SHS is often one of the health outcomes of interest in empirical analyses, as it is a valid predictor of morbidity and mortality [22]. In this modeling framework, we include economic deprivation through a control function approach that allows us to account for the correlation between the observable indicators and the employment status. We advise the reader that the present study is based on observational data and, therefore, it does not allow to interpret our results in terms of causal relations between SHS and covariates, but just in terms of association/correlation effects.

We provide novel empirical findings on the relationship between employment condition, economic deprivation and SHS. First, we are able to corroborate extant evidence on the negative effect of unemployment, precarious employment, and part-time employment on SHS. Second, we find that a more detailed definition of material deprivation and economic strain is able to partly account for the health effects of the employment status; nevertheless, we find that unemployment preserves a significant independent negative effect on SHS.

## 2. Methods

### 2.1. Data Source

The analysis is carried out on data from the third release of EU-SILC survey of Eurostat, including information on citizens from 26 European countries followed from 2009 to 2012 (Hungary and Croatia are excluded from the study due to the missing information about the employment status for year 2009). The original dataset is made of 127,199 individuals entered in the study in 2009; the 63.02% of these subjects (corresponding to 80,159 individuals) remains in the panel along the full time period 2009–2012. More precisely, from this balanced subsample we only consider individuals who were in working age (i.e., 17–64 years old) in the time period 2009–2012 and we drop all those subjects suffering from limitations in activities because of health problems or from any chronic (long-standing) illness as well as individuals who are unable to work in order to reduce the sample selection effect. Individuals who did not provide information about the type of job contract (open-ended vs. temporary) and individuals with missing values on one or more covariates of our interest are also dropped. The final total sample size amounts to 26,898 individuals and 107,592 observations (i.e., four measurements for each individual). Details on modalities of data collection, comparability of data between countries and over time, response rates, and any other question concerning the quality of data are provided by the official EU-SILC documentation freely available at http://ec.europa.eu/eurostat/web/income-and-living-conditions/overview.

SHS is described by an ordered polytomously-scored variable taking values 0 to 4 from *very poor* to *excellent* perceived health. As shown in Table 1 (first row), the great majority of respondents (92.34%) declares that his/her health status is good or excellent.

The employment status is a categorical variable defining different profiles in the labor market. We account for the duration of the contract by distinguishing between *permanent* employees, that is all dependent workers with an open-ended contract, and *temporary* workers, who are all employees with non-standard labor contracts including persons with a seasonal job, persons engaged by an employment agency and hired out to a third party to carry out a work mission (unless there is a work contract of unlimited duration with the employment agency or business), and persons with specific training contracts (see item PL140 of the EU-SILC questionnaire at http://ec.europa.eu/eurostat/web/income-and-living-conditions/overview).

We also consider a complementary definition for the labor market status according to the level of time effort and thereby discerning between *full-time* and *part-time* employees, alongside permanent and temporary employees. This definition allows us to compare the results of the empirical analysis with an alternative labor market profile that may capture a form of underemployment in terms of hours worked. Notice that, in this case, we are not able to prevent the bias from reverse causality to arise in our empirical analysis, as we do not have information on the reason for working fewer hours. The resulting classification allows us to distinguish four categories of employees: *full-time permanent* workers, *part-time permanent* workers, *full-time temporary* workers, and *part-time temporary* workers.

Individuals are defined as *unemployed* if they did not work during the year preceding the interview and are actively looking for a job. This definition of unemployment avoids that bias arises in the empirical analysis from the bidirectional nature of the relationship between SHS and unemployment: by considering only individuals looking for a job as unemployed, we are excluding the possibility of those persons that do not work because of bad health. The other modalities of the employment status distinguish between *self-employed*, that is, all self-employed workers with and without employees, and *other* individuals, which are inactive, such as students, home makers, retired workers, individuals in further training or unpaid work experiences, individuals in compulsory military or community service. In absence of further information, the last two categories can only be considered as residual in the empirical analysis since a reverse causality problem may emerge from workers that have chosen to be self-employed (with, for instance, more flexible hours compared to employees) or to stop working because of health limitations. Nevertheless, we keep these categories in our empirical analysis as their exclusion may result into a further selection bias problem.

The main interesting element arising from the distribution of SHS by employment categories concerns unemployed persons (Table 1): more than 11% of them declares an at most fair level of SHS. Instead, the distribution of SHS for *temporary* and *part-time* is very similar to that of *permanent* and *full-time* employees, respectively.

We observe some differences in the distribution of SHS by country and by the other covariates included in the analysis, as illustrated in Table 1. In particular, the 7.49% of total sample declares a fair level of SHS, however this percentage rises to 10.75% for citizens from Czech Republic, 13.93% for Poland, 18.87% for Portugal, 21.33% for Latvia, and 30.22% for Lithuania. Latvia and Lithuania are also those countries with the smallest percentage of persons with an excellent level of SHS: 7.74% and 7.10%, respectively, against the 36.54% of the total sample. Other countries with relatively few persons declaring an excellent level of SHS are Portugal (14.66%), Estonia (17.23%), Italy (21.99%), and Bulgaria (24.85%). On the other hand, countries with the best level of general self-perceived health are Cyprus (CY, 99.11% of interviewees evaluates as good or excellent the own health status), followed by The Netherlands (98.12%), Iceland (97.77%), Greece (97.74%), Spain (96.93%), Belgium (96.51%), Sweden (96.25%), United Kingdom (96.15%), and Austria (96.06%).

In the following inferential analysis, we control for the effect of the following set of covariates: household income per person, gender, age at baseline, year of interview (2009, 2010, 2011, 2012), education level (primary, secondary, tertiary), marital status (cohabitant on a legal basis, cohabitant without a legal basis, single). We also control for the unemployment rate (proportion of the labor force reporting unemployment) specific for each country and year (available at http://www.oecd.org/employment/labour-stats/), which provides a measure of relative deprivation, since the well-being of temporary workers and unemployed individuals may decrease less if they live in environments with a high unemployment rate [23]. From Table 1 we observe that young persons and males, with a higher income and a higher level of education, living by yourself or cohabitant without legal basis and living in countries with smaller levels of unemployment rates tend to have a better perception of their own general health status.

In order to account for economic deprivation, beyond the monetary household income, we include in the model specification a set of items available in the survey that refer to two main aspects related to economic deprivation: the economic strain and the material deprivation. The economic strain attains to a general financial distress and it is measured by the ability to make ends meet (with difficulty, with some difficulty, fairly easily, easily). The material deprivation encloses the following items, according to the definition provided by Eurostat (for details see at http://ec.europa.eu/eurostat/statistics-explained/index.php/Material_deprivation_statistics_-_early_results): presence of arrears on mortgages or rent payments, on utility bills, on hire purchase installments or other loans (no, yes), capacity to afford paying for one week holiday away from home (no, yes), capacity to afford a meal with meat, chicken, fish (or vegetarian equivalent) every second day (no, yes), capacity to face unexpected financial expenses (no, yes), possession of telephone, color TV, washing machine, car (yes, no-cannot afford, no-other reasons), and possession of an heating system to keep home adequately warm (no, yes). In addition to the mentioned items, we consider other three covariates that are available in the survey and refer to the presence of a leaking roof (no, yes), the level of financial burden of the total housing cost (heavy, slight, not burden at all), and the possession of a computer (yes, no-cannot afford, no-other reasons).

As shown in Table 2, the more impaired is the economic situation of a person, the worse his/her self-perceived health condition. For instance, the percentage of persons with an at most fair level of SHS decreases from 10.58% for those making ends meet with difficulty to 3.67% for those making easily ends meet. Similarly, for the items related to material deprivation the cumulative percentage of responses in categories *very poor*, *poor*, and *fair* is 4–7 percentage points lower for persons without economic deprivation compared to persons with an impaired economic status. Notice that we do not construct indicators for economic deprivation but just keep the detailed items provided by the EU-SILC. We do so in order to account for as much as the information on perceived and structural deprivation as available, while we do not infer on its direct interpretation, not being the focus of the present work.

Further, in order to explore the association between employment status and economic deprivation, we provide with Table 3 showing the conditional percentage distribution of employment status given the economic deprivation. Generally speaking, we observe a more impaired economic situation of unemployed individuals related to workers with permanent jobs. A significant association (Chi-square independence test with *p*-value less than 0.001) emerges with respect to all the economic deprivation variables taken into account. A particularly high association is related to ability to make ends meet (Cramer’s V = 0.1427), presence of arrears on utility bills (Cramer’s V = 0.1356), capacity to afford paying for one week holiday away from home (Cramer’s V = 0.2267), capacity to face unexpected financial expenses (Cramer’s V = 0.2042), possession of an heating system to keep home adequately warm (Cramer’s V = 0.1131), and level of financial burden of the total housing cost (Cramer’s V = 0.1364).

Other than the association between employment status and economic deprivation, data show that the distribution of the employment condition is also related to the country (Table 4). For instance, the percentage of full-time permanent workers ranges between 28.68% (Greece) and more than 74% (Norway and Denmark) and the percentage of part-time permanent workers ranges from 0.17% (Romania) to 30.25% (The Netherlands). Moreover, the percentage of full-time temporary workers is minimum for Estonia and UK (less than 1.00%) and maximum for Poland (12.10%), whereas Norway and The Netherlands show the smallest percentages of unemployed individuals (less than 1.00%) and Spain and Latvia the highest percentages (12.18% and 13.20%, respectively). The association between employment status and country reflects the different welfare systems characterizing each country. Indeed, aggregating countries by welfare regime, according to the classification provided in [24], a more homogeneous distribution of the employment status is observed within each group of countries (Table 4). We explicitly take into account the association at issue in our inferential analysis, according to the approach illustrated in Section 3.2.

Finally, it is worth to outline that, with the exception of country, gender and age at baseline, all the other variables are time-varying. However, for these variable we observe a high persistence of individuals in the modalities declared at the first year of interview. In particular, almost the 85% of interviewees remains in the same employment status over the period 2009–2012. We return on this point in the following section.

### 2.2. Statistical Analysis

The longitudinal structure of our dataset, with repeated measurements observed on a set of individuals along years 2009–2012, drives the choice of the statistical method for the data analysis to the class of random-effects (or multilevel or hierarchical) generalized linear models for panel data. In this section, we illustrate the class of models at issue; for more details see, among others, [25,26,27,28,29]. More in detail, due to the ordinal nature of the dependent variable, we estimate a random-effects ordered logit model [27,28,30,31].

Let $y_{it}$ be the observed ordered response variable SHS, assuming values y=0,1,2,3,4 for individual *i* at time *t*, with i=1,…,n and t=1,…,T. Moreover, let $x_{it}$ be a vector collecting the time-varying observed variables and $z_{i}$ a vector for the observed time-constant individual characteristics (e.g., country and gender).

The random-effects ordered logit model is formulated according to a link function based on global logits [32], as follows:

(1) $$\log\frac{p(y_{it}\geq y|u_{i},x_{it})}{p(y_{it}<y|u_{i},x_{it})}=\alpha_{0i}+x_{it}^{\prime }\beta ,\;i=1,\ldots ,n,\;t=1,\ldots ,T,$$

where $\alpha_{0i}$ is a subject-specific random intercept specified in terms of fixed and random parameters:

(2) $$\alpha_{0i}=\alpha_{0}+z_{i}^{\prime }\gamma +{\tilde{x}}_{i}^{\prime }\pi +\alpha_{i},\;i=1,\ldots ,n.$$

where $\alpha_{i}$ is a random parameter that summarizes the unobserved individual characteristics, which are time-constant and affect the probability of answering *y* across repeated measurements of SHS. As usual, the random effects $\alpha_{i}$ are assumed to be normally distributed with mean equal to 0 and constant variance $\sigma_{\alpha }^{2}$. We also consider the potential correlation between the employment status in $x_{it}$ and the individual unobserved heterogeneity, as the latter may non-randomly group workers into different labor market categories and bias their effect on SHS (for instance, a risky health-related life-style may also affect the worker’s employability). In order to tackle this issue, we employ a correlated random-effects approach [33,34,35], in which a parametric formulation of the time-constant correlation between the random effects and covariates of interest in $x_{it}$ is specified. Vector ${\tilde{x}}_{i}$ denotes a transformation of selected covariates in $x_{it}$, aimed at capturing the dependence between the corresponding elements in $x_{it}$ and $\alpha_{0i}$. In particular, ${\tilde{x}}_{i}$ includes four time-invariant covariates, one for each year in the sample, that represent the employment status of individual *i* in each year (for instance, if individual *i* is in employment condition $x_{1}$ in 2009, where $x_{1}$ is one of values taken by the employment condition covariate, then the first covariate we create will take value $x_{1}$ in all the four time occasions; if *i* is in condition $x_{2}$ in 2010, the second covariate will take value $x_{2}$ for the four time occasions, and so on). This is a standard strategy for correlated random effects when the variable of interest is qualitative [34]. Furthermore, we also consider the individual income by including in ${\tilde{x}}_{i}$ its average value, which represents a standard transformation for continuous covariates [35].

Substituting Equation (2) in Equation (1), the reduced-form of the random-effects ordered logit model is obtained:

(3) $$\log\frac{p(y_{it}\geq y|u_{i},x_{it},z_{i},{\tilde{x}}_{i})}{p(y_{it}<y|u_{i},x_{it},z_{i},{\tilde{x}}_{i})}=\alpha_{0}+x_{it}^{\prime }\beta +z_{i}^{\prime }\gamma +{\tilde{x}}_{i}^{\prime }\pi +\alpha_{i},\;i=1,\ldots ,n,\;t=1,\ldots ,T.$$

From Equation (3), it is clear that the probability of observing a given value of SHS depends both on the observed values of covariates in $x_{it}$, $z_{i}$, and ${\tilde{x}}_{i}$ and on the value assumed by the random component $\alpha_{i}$ for the *i*-th individual: values of $\alpha_{i}$ much higher (smaller) than zero imply a higher (smaller) level of SHS compared to the “average” individual, being constant all the observed covariates.

The model at issue is estimated through the marginal maximum log-likelihood approach, consisting in marginalizing out the distribution of the random effects and maximizing the resulting log-likelihood relative to the unknown model parameters. The integral involved in the log-likelihood function cannot be solved in closed form and it is approximated in a weighted sum, using the adaptive Gauss-Hermite quadrature method (for details see [27]).

In order to investigate whether economic deprivation is able to partly capture the effect of the employment condition on SHS, we adopt a control function approach [36] that accounts for the correlation between economic deprivation and employment status, under the assumption that the dependence between log-odds ratio in Equation (3) and the set of economic deprivation covariates has a linear form. In practice, we add to the set of explanatory variables the indicators of material deprivation and economic strain provided by the survey.

We stress that the approach we adopt in this paper is based on the assumption that the correlation between the employment status and the unobserved random effects has a linear parametric form, as opposed to the fixed-effects approach, in which the $\alpha_{i}$ are assumed to be fixed parameters, and therefore robust to violations of the implicit parametric assumption in Equation (2). Unfortunately, as mentioned at the very end of the previous section, we verified that the time-varying variables, *in primis* the one denoting the employment status (but also those denoting the economic deprivation), are quite persistent. Since the conditional maximum likelihood estimation method, on which the fixed-effects approach is based, only relies on the information provided by the time variation in the covariates, weak identification problems may arise in presence of highly persistent time-varying variables, since the Hessian of the log-likelihood may be close to being not negative definite [37]. Moreover, the fixed-effects approach does not identify the effects associated with time-constant covariates. A common solution to this problem consists in estimating a different model for each modality of each time-constant variable. In our study, we are specially interested in the effect of the country, which consists of 26 modalities. We also remind that formulating a specific model for each modality of a given time-constant variable corresponds to assuming interaction effects between the variable at issue and each other variable in the model, which is different from Model (3).

## 3. Results and Discussion

In this section we first illustrate the results related to two models specified according to Equation (3), which enclose all the covariates listed in Table 1 and differing one other by the presence of economic deprivation variables (listed in Table 2). Then, we group countries according to their welfare regime (see Table 3) and illustrate the main results obtained estimating Model (3) separately for each group. For each model, we report the estimates of odds ratios and corresponding standard errors, *z*-values and corresponding *p*-values, and confidence intervals at 95%; moreover, for the models related to the total sample of countries, we also provide the value of log-likelihood at convergence, the intraclass correlation coefficient (ICC), which denotes that part of total variance of the dependent variable explained by the longitudinal structure of data, the Akaike’s information criterion (AIC [38]), and the Bayesian information criterion (BIC [39]).

### 3.1. Analysis for All Countries

Table 5 refers to the estimated parameters for the model on the entire sample of countries and without controlling for economic deprivation. In addition to the individual covariates listed in Table 1, we also introduce a set of time-constant covariates to face the possible correlation between subject-specific random effects and the employment status (as illustrated in Section 2.2): the average individual income and the employment status observed for each year.

As concerns the employment status, employees with a part-time permanent work and those with a full-time temporary work have a highly significant disadvantage in SHS compared to full-time permanent workers (odds ratios 0.878 ± 0.101 and 0.889 ± 0.094, respectively). We also observe that part-time temporary work and full-time permanent work do not significantly differ from one other. Therefore, we conclude that part-time employment represents a disadvantage (in terms of SHS) compared to full-time employment, as well as temporary work compared to permanent work; however, there is no significant interaction effect between time effort and contract duration. In other words, the limited duration of a temporary job does not exacerbates the negative effect of a part-time job and, similarly, the less time effort of a part-time job does not worse the negative effect of a temporary job. Moreover, the odds ratio for unemployment (0.790 ± 0.089) denotes that unemployed individuals have a significantly lower propensity to evaluate in a satisfactorily way their own health status than full-time permanent employees. Note that the odds ratio for unemployed is sensibly lower than the odds ratios for part-time permanent and full-time temporary employees. This result is consistent with the findings that document the stronger adverse effect of unemployment on SHS compared to temporary employment. Indeed, the former has been found to exert detrimental effects also on physical aspects of SHS, through the engagement in unhealthy life-styles, whereas the latter is more associated with the psychological dimension of SHS. However, we advise the reader that we cannot definitely conclude for a significant difference between unemployed and the two types of employees, as the 95% univariate intervals for the corresponding regression parameters slightly overlap. We remind that, under the assumption of normality and equal variance (say, *σ*), the difference between two quantities (say, $\beta_{1}$ and $\beta_{2}$) is significant at 95% if the corresponding univariate intervals, whose inferior and superior limits are computed as $\beta_{h}\pm 1.96\sqrt{2}\sigma =\beta_{h}\pm 1.39\sigma$ (h=1,2), do not overlap [40]. In our case, we obtain the following intervals: [−0.208; −0.053] for the regression coefficient of part-time permanent workers, [−0.188; −0.047] for the regression coefficient of full-time temporary workers, and [−0.312; −0.160] for unemployed. Finally, the odds ratio estimated for the self-employed does not outline any specific advantage compared to full-time permanent workers.

Regarding the country-specific effects, we observe that the propensity to positively evaluate the SHS is definitely worse for almost all European citizens compared to Swedish people (i.e., the category of reference). In particular, citizens from Lithuania, Latvia, Portugal, Estonia, Italy, and Poland report very low odds ratios (superior limit of confidence intervals less than 0.20); on the contrary, no significant difference is observed for Austria, Cyprus, Romania, and United Kingdom, whereas citizens from Iceland and Greece have a higher propensity to positively evaluate the SHS compared to Swedish people.

Our estimation results also confirm the presence of significant effects associated with the major demographic and socio-economic determinants of SHS: there is a significant better perception of the level of SHS for subjects that are young, for males, for those having a secondary or tertiary education level, and for individuals with a higher average income. We find also that the marital status does not have a significant effect on the perceived SHS. It is worth noting that the four time-constant covariates describing the employment status for each year are globally statistically significant ($\chi_{4}^{2}=11.990$, *p*-value equal to 0.017), so contributing to capture a piece of correlation between employment status and SHS. Finally, the unemployment rate at the country-year level has a positive effect on SHS: this result is not uncommon in the empirical literature indicating that individuals’ SHS is better if they live in contexts where the economic deprivation is relatively high.

Using these results as benchmark, we now turn to testing whether material deprivation and economic strain are able to capture part of the negative effect of the employment condition on SHS. For this aim we include the indicators on material deprivation and economic strain provided by the EU-SILC survey in the model specification (Table 6). It is worth to outline that the model with deprivation indicators fits significantly better than the one without deprivation indicators (likelihood ratio test: deviance = 686.998, degrees of freedom = 23, *p*-value less than 0.0001).

After controlling for economic deprivation, the negative effects of part-time, temporary and unemployed conditions on the perceived health status are slightly reduced, as outlined by the increased values of odds ratios and of related *p*-values. In particular, only unemployment maintains a significant difference (*p*-value equal to 0.023) compared to the status of full-time permanent employees. This result suggests that the correlation between economic deprivation and employment condition, if neglected, may lead to overstate the magnitude of the effect of having a part-time or a temporary contract and being unemployed on SHS. At the same time, it appears that economic deprivation is able to partly capture the effect of the employment condition on SHS. Notice, however, that being the odds ratio of unemployed still statistically significant, the status of unemployment preserves an independent negative effect on health that is not channeled by its correlation with a condition of material deprivation and economic strain.

In addition, some of the indicators used to proxy economic deprivation also emerge as strong predictors of SHS, such as the ability to make ends meet and the level of material deprivation resulting by arrears on utility bills or loan payments, the capacity to afford a week holiday per year and an healthy meal every two days, and, more in general, the capacity to face unexpected financial expenses. Housing conditions, which are measured by the presence of an heating system to keep home adequately warm and by the presence of a leaking roof or other elements denoting situations of severe discomfort, have also a relevant role in affecting the perceived health status. Finally, as concerns other elements of economic deprivation, owning a washing machine affects significantly SHS, whereas no effect is due to owning telephone, color TV, car, and computer.

### 3.2. Analysis by Welfare Regime

We illustrate estimation results for subsets of countries grouped according to four different welfare regimes [24]: northern (Sweden, Denmark, Finland, Iceland, Norway), continental (Austria, Belgium, France, Luxembourg, The Netherlands), eastern (Bulgaria, Czech Republic, Estonia, Hungary, Latvia, Lithuania, Romania, Poland, Slovakia, Slovenia), and southern countries (Cyprus, Greece, Italy, Malta, Portugal, Spain); the Anglo-Saxon regime (including United Kingdom) is ignored due to the small sample size.

In Table 7, Table 8, Table 9 and Table 10, the estimates of odds ratios related to the employment status are shown for the two models without and with economic deprivation indicators (upper and bottom panels, respectively); estimates for the remaining covariates are not shown for the sake of parsimony.

Disentangling the sample by welfare regime, the negative effect of temporary employment appears irrelevant with the only exception of southern countries, where full-time temporary workers present a significant disadvantage compared to full-time permanent colleagues, even if after controlling for economic deprivation this disadvantage disappears (Table 10). By contrast, in northern and eastern countries a reduced time effort does not favor a positive perception of the health status (see odds ratios and related *p*-values for part-time permanent workers in Table 7 and Table 9) and this disadvantage persists also after controlling for material deprivation and economic strain. Finally, it is worth noticing that the unemployed status is completely irrelevant in northern and continental countries (Table 7 and Table 8, respectively) and partially irrelevant in southern countries (Table 10), whereas it preserves a significant adverse effect on SHS in eastern countries (Table 9) that is not explained by its correlation with material deprivation and economic strain.

These results are consistent with the patterns that emerge from the empirical evidence on the adverse effect of temporary and underemployment by welfare regime. Differences across welfare regimes are due to the fact that northern and continental countries benefit from an effective employment protection system and generous unemployment benefits, whereas southern and especially eastern European countries are not characterized by welfare provisions [24]. Also, it must be noted that the disaggregation into the four combinations of permanent vs temporary/full-time vs part-time jobs often results into a small number of workers per cell, which may prevent statistically significant effects from emerging.

## 4. Conclusions

In this paper we provide novel empirical findings on the relationship between employment condition, economic deprivation, and SHS. First, we are able to corroborate extant evidence on the negative association between the employment status, namely unemployment, precarious employment, and part-time employment, and SHS, adopting household monetary income as a measure of economic deprivation. Using this set of results as a benchmark, we then find that a more detailed definition of material deprivation and financial distress is partly able to capture the adverse correlation with precarious and part-time employment and of unemployment. Nevertheless, unemployment preserves a statistically significant, although weaker in magnitude, independent negative association with SHS. In this respect, our findings closely relate to very recent empirical evidence on unemployment, poverty, and SHS [6]. In addition to the mediating effect of deprivation indicators, it has to be outlined the relevant role played by the welfare regime. In particular, the SHS for part-time permanent workers in northern and eastern countries and for unemployed in southern countries appears significantly worse than the SHS for full-time permanent workers in the same countries.

Furthermore, it emerges that some of the indicators used to proxy economic deprivation are strong predictors of SHS, and that their correlation with the employment condition is such that it should not be neglected in empirical analysis, when available and further to the monetary income. The lack of information on involuntary part-time work does not allow us to infer on the effect of part-time working on SHS and, therefore, the evidence from this exercise can only be regarded as descriptive. In the traditional economic framework, where workers endogenously allocate work hours and leisure, part-time may actually be the optimal solution to the utility maximization problem. In such a case, the effect of part-time working on health is not necessarily negative. Nevertheless, the comparison of the estimation results with and without measures of economic deprivation provides similar insights.

The main limitation of this work is the incomplete identification of the effect of employment status on SHS, since we rely on a parametric assumption to model the correlation between the employment status and the unobserved individual characteristics that may non-randomly group workers into different categories and bias their effect on SHS. In order to account for time-constant unobserved heterogeneity, possibly correlated with the employment condition, a fixed-effects approach based on information on the transitions of the variables of interest can be adopted, so as to avoid parametric assumptions [5,41]. Unfortunately, the lack of time variation in our variables of interest does not allow us to pursue this kind of strategy. Nevertheless, the selection of the sample employed in the empirical analysis, based on excluding individuals that are affected by chronic diseases or suffer from limitations in activities because of health problems, and the definition of the status of unemployment, which accounts for individuals that did not work during the year preceding the interview and are actively looking for a job, allow us to avoid another source of bias, that is the possible reverse causation due to the bidirectional nature of the relationship between employment and health. In addition, the focus of the present work is on testing whether economic deprivation is able to channel part of the negative association between precarious employment and unemployment and SHS, whereas the mechanisms through which the employment status still exerts a significant effect on perceived health, possibly including the health selection mechanism, are not explored here and left for future research.

## Figures and Tables

**Table 1:** Descriptive statistics for SHS (%) by total sample, employment status, country, household income (in classes), gender, age at baseline (in classes), year of interview, education level, marital status.

General Health	Very Poor	Poor	Fair	Good	Excellent	Total
**Total**	0.02	0.15	7.49	55.80	36.54	100.00
**Employment status**						
Employees	0.01	0.11	7.11	57.94	34.82	59.58
*Full-time permanent*	*0.01*	*0.12*	*7.29*	*58.51*	*34.07*	*48.26*
*Part-time permanent*	*0.02*	*0.13*	*6.01*	*55.47*	*38.38*	*5.39*
*Full-time temporary*	*0.00*	*0.08*	*7.01*	*55.71*	*37.20*	*4.84*
*Part-time temporary*	*0.00*	*0.00*	*5.22*	*54.96*	*39.82*	*1.10*
Unemployed	0.03	0.52	10.63	57.12	31.70	5.78
Self employed	0.03	0.12	7.74	58.09	34.03	9.24
Other	0.05	0.18	7.67	50.98	41.11	25.39
**Country**						
Austria	0.06	0.09	3.79	35.33	60.72	2.89
Belgium	0.03	0.12	3.33	49.20	47.31	2.80
Bulgaria	0.00	0.42	9.48	65.26	24.85	5.59
Cyprus	0.16	0.00	0.73	29.34	69.76	2.13
Czech Republic	0.07	0.11	10.75	59.35	29.71	3.76
Denmark	0.00	0.10	4.80	48.47	46.63	0.85
Estonia	0.00	0.00	8.06	74.70	17.23	1.17
Finland	0.08	0.08	5.10	56.41	38.32	1.05
France	0.00	0.11	5.95	51.63	42.32	12.42
Greece	0.00	0.00	2.26	26.24	71.50	4.59
Iceland	0.00	0.12	2.11	30.28	67.49	0.74
Italy	0.08	0.10	6.06	71.76	21.99	7.12
Latvia	0.00	0.25	21.33	70.68	7.74	2.76
Lithuania	0.04	0.54	30.22	62.10	7.10	2.38
Luxembourg	0.00	0.24	6.31	51.51	41.94	7.54
Malta	0.00	0.03	6.02	63.77	30.19	3.43
The Netherlands	0.00	0.08	1.80	56.57	41.55	2.15
Norway	0.03	0.07	4.33	47.47	48.10	2.49
Poland	0.00	0.40	13.93	58.93	26.75	8.06
Portugal	0.06	0.15	18.87	66.26	14.66	2.91
Romania	0.00	0.00	4.42	55.88	39.70	5.66
Slovakia	0.02	0.09	6.07	58.16	35.66	4.80
Slovenia	0.19	0.09	8.73	58.02	32.97	1.83
Spain	0.01	0.07	2.99	68.70	28.23	7.59
Sweden	0.00	0.26	3.49	38.50	57.75	1.34
United Kingdom	0.00	0.04	3.81	38.10	58.05	1.95
**Household income**						
[0; 12, 228)	0.02	0.30	12.92	59.90	26.86	25.00
[12, 228; 26, 605)	0.02	0.13	8.24	57.85	33.76	25.00
[26, 605; 47, 930)	0.04	0.09	5.02	55.12	39.72	25.00
≥47,930	0.02	0.08	3.78	50.34	45.79	25.00
**Gender**						
Female	0.03	0.15	8.29	56.60	34.93	49.41
Male	0.02	0.15	6.67	54.99	38.18	50.59
**Age at baseline**						
[0, 29)	0.04	0.05	2.38	45.26	52.27	25.00
[29, 37)	0.02	0.14	4.96	55.47	39.41	25.00
[37, 48)	0.01	0.19	8.57	60.65	30.58	25.00
[48, 64]	0.02	0.24	14.69	62.57	22.49	25.00
**Year**						
2009	0.02	0.17	7.41	53.30	39.10	25.00
2010	0.01	0.11	6.96	55.96	36.96	25.00
2011	0.02	0.16	7.66	56.92	35.24	25.00
2012	0.05	0.16	7.92	57.02	34.85	25.00
**Education**						
Primary	0.03	0.18	9.45	59.09	31.25	23.24
Secondary	0.03	0.17	7.91	56.00	35.89	46.25
Tertiary	0.01	0.09	5.36	52.99	41.53	30.52
**Marital status**						
Cohab. with legal basis	0.02	0.16	9.16	59.52	31.14	52.59
Cohab. without legal basis	0.03	0.26	5.43	54.30	39.98	8.78
Single	0.03	0.11	5.68	51.08	43.09	38.63
**Unemployment rate**						
[3.20, 7.08)	0.03	0.20	5.90	51.76	42.15	25.00
[7.08, 9.15)	0.03	0.12	5.71	54.38	39.76	25.00
[9.15, 12.06)	0.02	0.18	8.85	56.92	34.04	25.00
[12.06, 24.94]	0.02	0.16	9.70	60.36	29.76	25.00

**Table 2:** Descriptive statistics for SHS (%) by economic deprivation status.

General Health	Very Poor	Poor	Fair	Good	Excellent	Total
**Ability to make ends meet**						
With difficulty	0.04	0.30	10.24	56.76	32.66	27.11
With some difficulty	0.02	0.12	8.73	59.07	32.06	31.35
Fairly easily	0.01	0.10	5.63	56.07	38.18	23.74
Easily	0.02	0.05	3.60	48.21	48.13	17.80
**Arrears on mortgage**						
No/owner/rent-free	0.02	0.14	7.49	55.81	36.53	96.18
Yes	0.02	0.32	7.44	55.48	36.74	3.82
**Arrears on utility bills**						
No	0.02	0.13	7.16	55.79	36.89	90.95
Yes	0.02	0.35	10.80	55.88	32.95	9.05
**Arrears on loan payments**						
No	0.03	0.13	7.45	56.02	36.37	96.84
Yes	0.00	0.66	8.70	49.12	41.52	3.16
**Annual holiday**						
No	0.02	0.27	10.72	58.82	30.17	36.53
Yes	0.02	0.08	5.63	54.07	40.20	63.47
**Meal with meat, chicken, fish**						
No	0.01	0.54	13.81	60.49	25.15	9.13
Yes	0.03	0.11	6.85	55.33	37.68	90.87
**Unexpected financial exp.**						
No	0.04	0.30	10.94	59.10	29.61	31.79
Yes	0.02	0.08	5.88	54.26	39.76	68.21
**Telephone**						
Yes	0.02	0.15	7.43	55.78	36.61	98.69
No-cannot afford	0.00	0.60	11.24	58.13	30.02	0.72
No-other reasons	0.00	0.15	12.28	55.77	31.80	0.58
**Color TV**						
Yes	0.02	0.15	7.48	55.88	36.46	98.77
No-cannot afford	0.00	0.00	12.27	61.73	25.99	0.24
No-other reasons	0.00	0.09	7.11	46.75	46.05	1.00
**Washing machine**						
Yes	0.02	0.15	7.41	55.79	36.62	97.55
No-cannot afford	0.00	0.31	9.87	59.62	30.19	1.37
No-other reasons	0.00	0.16	11.39	51.72	36.73	1.08
**Car**						
Yes	0.03	0.11	6.77	55.54	37.56	85.71
No-cannot afford	0.00	0.45	11.27	58.50	29.79	8.45
No-other reasons	0.01	0.37	12.57	55.76	31.28	5.84
**Heating to keep home adequately warm**						
No	0.02	0.40	13.29	59.59	26.69	10.25
Yes	0.02	0.12	6.81	55.37	37.66	89.75
**Leaking roof**						
No	0.02	0.11	7.41	55.47	37.33	86.92
Yes	0.03	0.33	10.27	58.02	31.34	13.08
**Financial burden of housing costs**						
Heavy	0.04	0.25	9.03	58.28	32.40	33.65
Slight	0.02	0.11	7.41	56.61	35.85	46.67
Not at all	0.01	0.09	5.05	49.63	45.23	19.68
**Computer**						
Yes	0.02	0.13	6.58	55.26	38.01	85.14
No-cannot afford	0.03	0.18	11.54	59.35	28.89	5.74
No-other reasons	0.03	0.33	13.44	58.65	27.55	9.13

**Table 3:** Conditional percentage distribution of employment status (%) given economic deprivation status.

Employment Status	Full-Time Permanent	Part-Time Permanent	Full-Time Temporary	Part-Time Temporary	Unemployed	Self-Employed	Other
**Ability to make ends meet**							
With difficulty	37.69	2.83	5.62	1.11	12.40	8.75	31.61
With some difficulty	49.07	4.06	5.12	1.07	5.16	9.63	25.88
Fairly easily	53.78	6.38	4.56	1.19	2.59	9.25	22.25
Easily	55.11	10.14	3.58	0.99	1.35	9.28	19.56
**Arrears on mortgage**							
No/owner/rent-free	48.72	5.47	4.81	1.08	5.52	9.16	25.24
Yes	36.16	3.37	5.55	1.39	12.75	11.41	29.37
**Arrears on utility bills**							
No	49.50	5.70	4.84	1.10	4.98	9.06	24.81
Yes	35.34	2.19	4.82	1.01	14.12	11.08	31.44
**Arrears on loan payments**							
No	48.68	5.47	4.81	1.08	5.51	9.13	25.32
Yes	35.01	2.81	5.88	1.48	14.28	12.73	27.80
**Annual holiday**							
No	38.98	2.64	5.98	1.08	10.82	9.17	31.34
Yes	53.43	6.92	4.21	1.11	2.98	9.28	22.08
**Meal with meat, chicken, fish**							
No	36.80	2.38	5.13	0.72	14.35	7.63	32.99
Yes	49.38	5.68	4.81	1.13	4.94	9.40	24.65
**Unexpected financial exp.**							
No	40.51	3.52	5.96	1.24	11.52	7.31	29.95
Yes	51.82	6.25	4.33	1.03	3.15	10.13	23.30
**Telephone**							
Yes	48.47	5.43	4.84	1.10	5.69	9.16	25.31
No-cannot afford	23.21	0.92	4.27	0.61	17.86	20.46	32.67
No-other reasons	40.28	3.68	4.73	0.70	8.41	11.03	31.17
**Color TV**							
Yes	48.31	5.37	4.83	1.09	5.77	9.21	25.42
No-cannot afford	27.40	1.83	4.11	1.83	13.70	12.79	38.36
No-other reasons	48.02	7.81	6.42	1.88	5.04	11.36	19.47
**Washing machine**							
Yes	48.48	5.45	4.87	1.10	5.72	9.09	25.30
No-cannot afford	29.58	1.74	2.40	0.83	11.52	22.37	31.57
No-other reasons	48.38	4.17	4.91	1.20	5.56	8.62	27.15
**Car**							
Yes	49.24	5.84	4.81	1.11	4.97	9.56	24.47
No-cannot afford	41.31	2.04	4.78	0.82	13.29	6.98	30.78
No-other reasons	43.64	3.39	5.42	1.25	7.19	7.64	31.47
**Heating to keep home adequately warm**							
No	41.56	2.01	5.16	1.09	12.40	8.04	29.74
Yes	49.00	5.76	4.80	1.10	5.05	9.37	24.91
**Leaking roof**							
No	49.31	5.38	4.70	1.05	5.31	9.19	25.06
Yes	41.25	5.48	5.75	1.39	8.96	9.55	27.61
**Financial burden of housing costs**							
Heavy	41.03	3.91	5.84	1.19	10.04	8.23	29.76
Slight	50.73	4.82	4.45	0.92	4.18	10.33	24.56
Not at all	54.71	9.25	4.04	1.35	2.33	8.38	19.94
**Computer**							
Yes	49.65	5.92	4.89	1.14	5.01	8.82	24.57
No-cannot afford	36.52	1.38	4.81	0.92	15.62	10.70	30.05
No-other reasons	42.07	2.69	4.39	0.73	7.21	12.45	30.46

**Table 4:** Conditional percentage distribution of employment status given country and welfare regime.

Employment Status	Full-Time Permanent	Part-Time Permanent	Full-Time Temporary	Part-Time Temporary	Unemployed	Self-Employed	Other
**Anglo-Saxon**	*35.26*	*10.42*	*0.77*	*0.77*	*3.35*	*19.05*	*30.37*
United Kingdom	35.26	10.42	0.77	0.77	3.35	19.05	30.37
**Northern**	*66.82*	*8.15*	*3.12*	*0.83*	*1.93*	*6.74*	*12.41*
Denmark	74.31	9.38	1.50	0.69	2.43	1.27	10.42
Finland	50.15	3.56	2.87	0.30	2.67	20.47	19.98
Iceland	53.99	6.73	3.99	0.87	3.12	10.10	21.20
Norway	74.82	6.63	3.00	0.70	0.63	5.66	8.55
Sweden	66.10	14.69	4.08	1.56	2.97	0.07	10.53
**Continental**	*48.49*	*12.52*	*4.17*	*1.94*	*3.44*	*6.74*	*22.71*
Austria	50.08	14.95	2.33	0.99	1.81	7.85	21.98
Belgium	44.15	17.05	2.79	1.63	3.49	6.15	24.73
France	49.47	8.79	5.70	2.49	4.59	6.40	22.55
Luxembourg	50.85	10.73	3.10	1.16	2.96	5.86	25.34
The Netherlands	38.45	30.25	3.43	3.22	0.71	10.91	13.04
**Eastern**	*51.96*	*1.05*	*5.12*	*0.45*	*6.34*	*9.42*	*25.67*
Bulgaria	59.54	0.65	1.69	0.18	6.53	6.82	24.60
Czech Republic	59.11	0.77	5.83	0.24	3.34	11.21	19.50
Estonia	61.54	3.37	0.32	0.16	8.01	6.41	20.19
Latvia	55.12	2.60	2.06	0.22	13.20	3.11	23.69
Lithuania	63.07	2.17	1.25	0.04	10.66	5.07	17.74
Poland	37.65	1.08	12.10	1.25	6.18	14.02	27.72
Romania	54.09	0.17	1.14	0.16	2.79	12.29	29.36
Slovakia	50.64	0.68	5.38	0.19	7.30	7.18	28.63
Slovenia	51.24	1.14	5.64	0.64	4.45	6.73	30.17
**Southern**	*38.95*	*2.89*	*5.71*	*1.14*	*8.50*	*11.98*	*30.83*
Cyprus	48.44	1.96	6.78	0.94	4.73	5.46	31.68
Greece	28.68	1.17	4.14	1.10	10.08	23.96	30.86
Italy	36.45	4.59	4.08	1.20	6.96	11.74	35.00
Malta	40.27	3.03	2.23	0.42	2.78	8.56	42.71
Portugal	49.81	0.96	9.93	0.48	8.90	10.13	19.79
Spain	39.73	3.11	7.53	1.68	12.18	9.48	26.29

**Table 5:** Correlated random-effects ordered logit model *without economic deprivation*—All countries (Log-likelihood = −72,016.849; ICC = 0.538; AIC = 144,129.70; BIC = 144,523.29).

	Odds Ratio	St. Err.	*z*-Value	*p*-Value	Inf_95%_	Sup_95%_
Employment status (ref.: Full-time permanent)						
Part-time permanent	0.878	0.049	−2.330	0.020	0.787	0.979
Full-time temporary	0.889	0.045	−2.310	0.021	0.804	0.983
Part-time temporary	0.929	0.093	−0.730	0.465	0.763	1.131
Unemployed	0.790	0.043	−4.330	0.000	0.710	0.879
Self-employed	1.068	0.084	0.840	0.402	0.916	1.245
Other	0.937	0.049	−1.230	0.218	0.845	1.039
Country (ref.: Sweden)						
Austria	1.068	0.167	0.420	0.675	0.786	1.451
Belgium	0.500	0.078	−4.460	0.000	0.369	0.678
Bulgaria	0.269	0.041	−8.620	0.000	0.200	0.363
Cyprus	1.321	0.217	1.700	0.089	0.958	1.822
Czech Republic	0.239	0.036	−9.500	0.000	0.178	0.321
Denmark	0.536	0.109	−3.060	0.002	0.360	0.799
Estonia	0.101	0.019	−12.240	0.000	0.070	0.146
Greece	3.634	0.574	8.180	0.000	2.667	4.951
Finland	0.231	0.045	−7.560	0.000	0.158	0.337
France	0.296	0.040	−8.960	0.000	0.227	0.386
Iceland	1.813	0.391	2.760	0.006	1.189	2.766
Italy	0.102	0.014	−16.220	0.000	0.077	0.134
Latvia	0.031	0.005	−20.540	0.000	0.022	0.043
Lithuania	0.024	0.004	−22.040	0.000	0.018	0.034
Luxembourg	0.237	0.034	−10.180	0.000	0.180	0.313
Malta	0.218	0.034	−9.700	0.000	0.161	0.297
The Netherlands	0.456	0.074	−4.850	0.000	0.333	0.627
Norway	0.436	0.070	−5.200	0.000	0.319	0.596
Poland	0.149	0.022	−13.180	0.000	0.112	0.198
Portugal	0.043	0.007	−19.750	0.000	0.032	0.059
Romania	0.924	0.145	−0.510	0.612	0.680	1.255
Slovakia	0.220	0.033	−10.140	0.000	0.164	0.294
Slovenia	0.150	0.025	−11.480	0.000	0.108	0.207
Spain	0.150	0.023	−12.330	0.000	0.111	0.203
United Kingdom	0.778	0.165	−1.180	0.237	0.513	1.179
Unempl. country-by-year	1.015	0.005	3.100	0.002	1.006	1.025
Log-income	1.047	0.031	1.540	0.124	0.987	1.111
Female	0.828	0.025	−6.240	0.000	0.780	0.878
Age at baseline	1.072	0.012	5.960	0.000	1.048	1.096
Squared age	1.015	0.005	3.100	0.002	1.006	1.025
Education (ref.: primary)						
secondary	1.206	0.042	5.330	0.000	1.126	1.292
tertiary	1.647	0.068	12.000	0.000	1.518	1.787
Marital status (ref.: cohab. with legal basis)						
cohab. without legal basis	0.924	0.044	−1.660	0.096	0.842	1.014
single	1.070	0.040	1.840	0.066	0.996	1.151
Year (ref.: 2009)						
2010	0.990	0.022	−0.460	0.647	0.948	1.033
2011	0.988	0.022	−0.570	0.567	0.946	1.031
2012	0.968	0.021	−1.500	0.133	0.927	1.010
Var. to control correlated random-effects						
Avg. income	1.672	0.069	12.510	0.000	1.542	1.812
Emp. 2009	1.033	0.021	1.600	0.110	0.993	1.074
Emp. 2010	1.039	0.025	1.610	0.108	0.992	1.089
Emp. 2011	0.943	0.022	−2.460	0.014	0.900	0.988
Emp. 2012	1.012	0.021	0.580	0.562	0.972	1.053
Threshold 1	−11.955	0.253	−47.210	0.000	−12.451	−11.459
Threshold 2	−9.894	0.174	−56.870	0.000	−10.235	−9.553
Threshold 3	−4.846	0.151	−32.160	0.000	−5.142	−4.551
Threshold 4	0.618	0.149	4.150	0.000	0.326	0.910
${\hat{\sigma }}_{\alpha }^{2}$	3.831	0.066			3.704	3.962

**Table 6:** Correlated random-effects ordered logit model for *with economic deprivation*—All countries (Log-likelihood = −71,673.350 ; ICC = 0.532; AIC = 143,488.7; BIC = 144,070.89).

	Odds Ratio	St. Err.	*z*-Value	*p*-Value	Inf_95%_	Sup_95%_
Employment status (ref.: Full-time permanent)						
Part-time permanent	0.900	0.050	−1.900	0.058	0.807	1.004
Full-time temporary	0.913	0.047	−1.780	0.075	0.826	1.009
Part-time temporary	0.987	0.099	−0.130	0.898	0.811	1.202
Unemployed	0.883	0.048	−2.270	0.023	0.793	0.983
Self-employed	1.081	0.085	0.990	0.323	0.927	1.260
Other	0.984	0.052	−0.320	0.752	0.887	1.090
Country (ref.: Sweden)						
Austria	1.232	0.192	1.340	0.181	0.908	1.671
Belgium	0.564	0.087	−3.720	0.000	0.417	0.763
Bulgaria	0.377	0.058	−6.380	0.000	0.279	0.509
Cyprus	2.198	0.362	4.780	0.000	1.592	3.037
Czech Republic	0.277	0.042	−8.520	0.000	0.206	0.372
Denmark	0.561	0.113	−2.870	0.004	0.377	0.833
Estonia	0.117	0.022	−11.510	0.000	0.081	0.169
Finland	5.085	0.806	10.260	0.000	3.727	6.937
France	0.252	0.049	−7.150	0.000	0.173	0.368
Greece	0.367	0.050	−7.410	0.000	0.281	0.479
Iceland	2.241	0.480	3.770	0.000	1.473	3.412
Italy	0.149	0.021	−13.480	0.000	0.113	0.196
Latvia	0.038	0.006	−19.480	0.000	0.027	0.052
Lithuania	0.031	0.005	−20.560	0.000	0.023	0.044
Luxembourg	0.296	0.042	−8.620	0.000	0.224	0.390
Malta	0.282	0.044	−8.060	0.000	0.207	0.384
The Netherlands	0.449	0.072	−4.990	0.000	0.328	0.615
Norway	0.472	0.075	−4.740	0.000	0.346	0.644
Poland	0.181	0.026	−11.840	0.000	0.137	0.240
Portugal	0.055	0.009	−18.260	0.000	0.040	0.075
Romania	1.005	0.157	0.030	0.977	0.739	1.366
Slovakia	0.271	0.040	−8.750	0.000	0.202	0.363
Slovenia	0.210	0.035	−9.470	0.000	0.152	0.290
Spain	0.193	0.030	−10.720	0.000	0.143	0.260
United Kingdom	0.981	0.207	−0.090	0.928	0.648	1.484
Log-income	0.989	0.030	−0.360	0.718	0.932	1.049
Female	0.833	0.025	−6.080	0.000	0.785	0.883
Age at baseline	0.431	0.006	−56.930	0.000	0.419	0.444
Squared age	1.060	0.012	5.070	0.000	1.036	1.084
Education (ref.: primary)						
secondary	1.124	0.040	3.310	0.001	1.049	1.204
tertiary	1.430	0.060	8.530	0.000	1.317	1.553
Marital status (ref.: cohab. with legal basis)						
cohab. without legal basis	0.933	0.044	−1.470	0.143	0.850	1.024
single	1.089	0.040	2.300	0.022	1.013	1.171
Unempl. country-by-year	1.016	0.005	3.280	0.001	1.006	1.026
Year (ref.: 2009)						
2010	0.990	0.022	−0.45	0 0.650	0.948	1.034
2011	0.988	0.022	−0.57	0 0.570	0.946	1.031
2012	0.967	0.021	−1.52	0 0.128	0.926	1.010
Var. to control correlated random-effects						
Avg. income	1.429	0.060	8.520	0.000	1.316	1.551
Emp. 2009	1.033	0.021	1.640	0.100	0.994	1.075
Emp. 2010	1.034	0.025	1.400	0.162	0.987	1.083
Emp. 2011	0.940	0.022	−2.620	0.009	0.897	0.985
Emp. 2012	1.007	0.020	0.330	0.739	0.968	1.047
Ability to make ends meet (ref.: with difficulty)						
with some difficulty	0.921	0.027	−2.770	0.006	0.869	0.976
fairly easily	1.082	0.041	2.080	0.037	1.005	1.165
easily	1.395	0.063	7.360	0.000	1.276	1.524
Arrears on mortgage	0.999	0.058	−0.020	0.985	0.891	1.119
Arrears on utility bills	0.891	0.036	−2.810	0.005	0.823	0.966
Arrears on loan payments	0.883	0.052	−2.100	0.036	0.786	0.992
Annual holiday	1.186	0.034	5.990	0.000	1.122	1.254
Meal with meat, chicken, fish	1.163	0.047	3.710	0.000	1.074	1.259
Unexpected financial exp.	1.139	0.032	4.600	0.000	1.077	1.203
Telephone (ref.: Yes)						
No-cannot afford	1.153	0.160	1.030	0.303	0.879	1.513
No-other reasons	1.096	0.140	0.720	0.472	0.853	1.409
Color TV (ref.: Yes)						
No-cannot afford	0.791	0.162	−1.140	0.253	0.529	1.182
No-other reasons	0.983	0.113	−0.150	0.884	0.785	1.232
Washing machine (ref.: Yes)						
No-cannot afford	1.281	0.148	2.150	0.032	1.022	1.606
No-other reasons	1.149	0.125	1.280	0.200	0.929	1.423
Car (ref.: Yes)						
No-cannot afford	0.935	0.046	−1.370	0.171	0.849	1.029
No-other reasons	0.893	0.046	−2.210	0.027	0.808	0.987
Heating	1.250	0.047	5.870	0.000	1.160	1.347
Leaking roof	0.743	0.023	−9.730	0.000	0.700	0.789
Financial burden of housing costs (ref.: A heavy burden)						
A slight burden	1.030	0.026	1.190	0.233	0.981	1.081
Not burden at all	1.228	0.045	5.590	0.000	1.142	1.319
Computer (ref.: Yes)						
No-cannot afford	0.929	0.050	−1.390	0.165	0.836	1.031
No-other reasons	0.861	0.037	−3.540	0.000	0.792	0.935
Threshold 1	−11.447	0.258	−44.390	0.000	−11.952	−10.941
Threshold 2	−9.384	0.181	−51.960	0.000	−9.738	−9.030
Threshold 3	−4.326	0.158	−27.300	0.000	−4.637	−4.015
Threshold 4	1.147	0.157	7.300	0.000	0.840	1.455
${\hat{\sigma }}_{\alpha }^{2}$	3.736	0.065			3.612	3.865

**Table 7:** Correlated random-effects ordered logit model: estimated odds ratios for employment status (ref.: Full-time permanent)—Northern countries.

	Odds Ratio	St. Err.	*z*-Value	*p*-Value	Inf_95%_	Sup_95%_
*Without economic deprivation*						
Part-time permanent	0.697	0.119	−2.110	0.035	0.499	0.975
Full-time temporary	0.766	0.167	−1.220	0.222	0.499	1.175
Part-time temporary	0.485	0.192	−1.830	0.068	0.223	1.055
Unemployed	0.852	0.252	−0.540	0.588	0.477	1.520
Self-employed	0.923	0.293	−0.250	0.800	0.495	1.720
Other	0.883	0.181	−0.610	0.543	0.590	1.320
*With economic deprivation*						
Part-time permanent	0.704	0.121	−2.040	0.041	0.503	0.986
Full-time temporary	0.768	0.169	−1.200	0.229	0.499	1.182
Part-time temporary	0.509	0.204	−1.690	0.092	0.232	1.116
Unemployed	0.917	0.274	−0.290	0.771	0.510	1.647
Self-employed	0.933	0.299	−0.220	0.829	0.498	1.748
Other	0.935	0.194	−0.330	0.745	0.622	1.404

**Table 8:** Correlated random-effects ordered logit model: estimated odds ratios for employment status (ref.: Full-time permanent)—Continental countries.

	Odds Ratio	St. Err.	*z*-Value	*p*-Value	Inf_95%_	Sup_95%_
*Without economic deprivation*						
Part-time permanent	0.982	0.073	−0.240	0.809	0.849	1.137
Full-time temporary	0.972	0.097	−0.290	0.774	0.800	1.181
Part-time temporary	1.028	0.144	0.200	0.842	0.781	1.354
Unemployed	0.875	0.096	−1.220	0.224	0.705	1.085
Self-employed	1.178	0.182	1.060	0.290	0.870	1.596
Other	1.186	0.117	1.730	0.083	0.978	1.439
*With economic deprivation*						
Part-time permanent	1.013	0.075	0.170	0.867	0.875	1.171
Full-time temporary	0.997	0.099	−0.030	0.976	0.821	1.211
Part-time temporary	1.084	0.152	0.570	0.567	0.824	1.426
Unemployed	0.994	0.110	−0.050	0.958	0.801	1.234
Self-employed	1.205	0.186	1.210	0.227	0.890	1.632
Other	1.247	0.123	2.240	0.025	1.028	1.513

**Table 9:** Correlated random-effects ordered logit model: estimated odds ratios for employment status (ref.: Full-time permanent)—Eastern countries.

	Odds Ratio	St. Err.	*z*-Value	*p*-Value	Inf_95%_	Sup_95%_
*Without economic deprivation*						
Part-time permanent	0.487	0.091	−3.860	0.000	0.338	0.702
Full-time temporary	0.967	0.090	−0.360	0.720	0.806	1.160
Part-time temporary	0.980	0.285	−0.070	0.943	0.554	1.733
Unemployed	0.683	0.067	−3.890	0.000	0.564	0.827
Self-employed	1.093	0.160	0.610	0.542	0.821	1.455
Other	0.771	0.074	−2.730	0.006	0.640	0.929
*With economic deprivation*						
Part-time permanent	0.504	0.094	−3.680	0.000	0.350	0.726
Full-time temporary	0.995	0.092	−0.050	0.956	0.829	1.194
Part-time temporary	1.095	0.320	0.310	0.756	0.618	1.940
Unemployed	0.780	0.077	−2.520	0.012	0.643	0.946
Self-employed	1.075	0.157	0.490	0.622	0.807	1.431
Other	0.810	0.077	−2.200	0.028	0.672	0.977

**Table 10:** Correlated random-effects ordered logit model: estimated odds ratios for employment status (ref.: Full-time permanent)—Southern countries.

	Odds Ratio	St. Err.	*z*-Value	*p*-Value	Inf_95%_	Sup_95%_
*Without economic deprivation*						
Part-time permanent	0.907	0.103	−0.870	0.385	0.726	1.131
Full-time temporary	0.828	0.066	−2.360	0.018	0.708	0.969
Part-time temporary	0.996	0.162	−0.030	0.979	0.724	1.370
Unemployed	0.827	0.073	−2.160	0.031	0.695	0.983
Self-employed	1.070	0.139	0.520	0.602	0.830	1.380
Other	0.962	0.090	−0.420	0.678	0.801	1.155
*With economic deprivation*						
Part-time permanent	0.920	0.104	−0.740	0.459	0.736	1.148
Full-time temporary	0.859	0.069	−1.890	0.058	0.735	1.005
Part-time temporary	1.055	0.172	0.330	0.744	0.766	1.453
Unemployed	0.907	0.081	−1.090	0.276	0.762	1.081
Self-employed	1.113	0.145	0.820	0.413	0.862	1.436
Other	0.999	0.094	−0.010	0.993	0.831	1.201

## Abbreviations

The following abbreviations are used in this manuscript:

SHS	Self-evaluated Health Status
EU-SILC	European Union Statistics on Income and Living Conditions
AIC	Akaike’s Information Criterion
BIC	Bayesian Information Criterion

## References

[1] Norström F., Virtanen P., Hammarström A., Gustafsson P.E., Janlert U. (2014). How does unemployment affect self-assessed health? A systematic review focusing on subgroup effects. BMC Public Health.

[2] Benach J., Vives A., Amable M., Vanroelen C., Tarafa G., Muntaner C. (2014). Precarious Employment: Understanding an Emerging Social Determinant of Health. Annu. Rev. Public Health.

[3] Friedland D.S., Price R.H. (2003). Underemployment: Consequences for the Health and Well-Being of Workers. Am. J. Community Psychol..

[4] OECD (2016). Stat Extracts. http://www.oecd.org/statistics/.

[5] Tøge A.G., Blekesaune M. (2015). Unemployment transitions and self-rated health in Europe: A longitudinal analysis of EU-SILC from 2008 to 2011. Soc. Sci. Med..

[6] Vaalavuo M. (2016). Deterioration in health: What is the role of unemployment and poverty?. Scand. J. Public Health.

[7] Barr B., Kinderman P., Whitehead M. (2015). Trends in mental health inequalities in England during a period of recession, austerity and welfare reform 2004 to 2013. Soc. Sci. Med..

[8] Brydsten A., Hammarström A., San Sebastian M. (2016). The impact of economic recession on the association between youth unemployment and functional somatic symptoms in adulthood: A difference-in-difference analysis from Sweden. BMC Public Health.

[9] Lundin A., Falkstedt D., Lundberg I., Hemmingsson T. (2014). Unemployment and coronary heart disease among middle-aged men in Sweden: 39,243 men followed for 8 years. Occup. Environ. Med..

[10] Roelfs D.J., Shor E., Davidson K.W., Schwartz J.E. (2011). Losing life and livelihood: A systematic review and meta-analysis of unemployment and all-cause mortality. Soc. Sci. Med..

[11] McKee-Ryan F., Song Z., Wanberg C.R., Kinicki A.J. (2005). Psychological and physical well-being during unemployment: A meta-analytic study. J. Appl. Psychol..

[12] Milner A., Page A., LaMontagne A.D. (2013). Long-term unemployment and suicide: A systematic review and meta-analysis. PLoS ONE.

[13] Vives A., Amable M., Ferrer M., Moncada S., Llorens C., Muntaner C., Benavides F.G., Benach J. (2013). Employment Precariousness and Poor Mental Health: Evidence from Spain on a New Social Determinant of Health. J. Environ. Public Health.

[14] Kim M.H., Kim C.y., Park J.K., Kawachi I. (2008). Is precarious employment damaging to self-rated health? Results of propensity score matching methods, using longitudinal data in South Korea. Soc. Sci. Med..

[15] Rugulies R., Aust B., Burr H., Bültmann U. (2008). Job insecurity, chances on the labour market and decline in self-rated health in a representative sample of the Danish workforce. J. Epidemiol. Community Health.

[16] Nishikitani M., Tsurugano S., Inoue M., Yano E. (2012). Effect of unequal employment status on workers’ health: Results from a Japanese national survey. Soc. Sci. Med..

[17] Waenerlund A.K., Virtanen P., Hammarström A. (2011). Is temporary employment related to health status? Analysis of the Northern Swedish Cohort. Scand. J. Public Health.

[18] Dawson C., Veliziotis M., Pacheco G., Webber D.J. (2015). Is temporary employment a cause or consequence of poor mental health? A panel data analysis. Soc. Sci. Med..

[19] Angrave D., Charlwood A. (2015). What is the relationship between long working hours, over-employment, under-employment and the subjective well-being of workers? Longitudinal evidence from the UK. Hum. Relat..

[20] Milner A., Smith P., LaMontagne A. (2015). Working hours and mental health in Australia: Evidence from an Australian population-based cohort, 2001–2012. Occup. Environ. Med..

[21] Rosenthal L., Carroll-Scott A., Earnshaw V.A., Santilli A., Ickovics J.R. (2012). The importance of full-time work for urban adults’ mental and physical health. Soc. Sci. Med..

[22] Schnittker J., Bacak V. (2014). The increasing predictive validity of self-rated health. PLoS ONE.

[23] Clark A.E. (2003). Unemployment as a social norm: Psychological evidence from panel data. J. Labor Econ..

[24] Kim I.H., Muntaner C., Vahid Shahidi F., Vives A., Vanroelen C., Benach J. (2012). Welfare states, flexible employment, and health: A critical review. Health Policy.

[25] Diggle P.J., Heagerty P., Liang K.Y., Zeger S.L. (2002). Analysis of Longitudinal Data.

[26] Goldstein G. (2003). Multilevel Statistical Models.

[27] Skrondal A., Rabe-Hesketh S. (2004). Generalized Latent Variable Modeling: Multilevel, Longitudinal, and Structural Equation Models.

[28] Snijders T.A.B., Bosker R.J. (2012). Multilevel Analysis. An Introduction to Basic and Advanced Multilevel Modeling.

[29] Verbeke G., Molenberghs G. (2000). Linear Mixed Models for Longitudinal Data.

[30] McCulloch C.E., Searle S.R., Neuhaus J.M. (2008). Generalized, Linear, and Mixed Models.

[31] Stroup W.W. (2012). Generalized Linear Mixed Models: Modern Concepts, Methods and Applications.

[32] Agresti A. (2002). Categorical Data Analysis.

[33] Wooldridge J.M. (2010). Econometric Analysis of Cross Section and Panel Data.

[34] Chamberlain G. (1980). Analysis of Covariance with Qualitative Data. Rev. Econ. Stud..

[35] Mundlak Y. (1978). On the Pooling of Time Series and Cross Section Data. Econometrica.

[36] Heckman J.J., Hotz V.J. (1989). Choosing among alternative nonexperimental methods for estimating the impact of social programs: The case of manpower training. J. Am. Stat. Assoc..

[37] Hsiao C. (2005). Analysis of Panel Data.

[38] Akaike H., Petrov B.N., Csaki F. (1973). Information theory and an extension of the maximum likelihood principle. Second International Symposium of Information Theory.

[39] Schwarz G. (1978). Estimating the Dimension of a Model. Ann. Stat..

[40] Goldstein H., Healy M.J.R. (1995). The graphical presentation of a collection of means. J. R. Stat. Soc. Ser. A.

[41] Minelli L., Pigini C., Chiavarini M., Bartolucci F. (2014). Employment status and perceived health condition: Longitudinal data from Italy. BMC Public Health.

